# Clinical Manifestations of Bacteremia Caused by *Aeromonas* Species in Southern Taiwan

**DOI:** 10.1371/journal.pone.0091642

**Published:** 2014-03-10

**Authors:** Hung-Jen Tang, Chih-Cheng Lai, Hsin-Lan Lin, Chien-Ming Chao

**Affiliations:** 1 Department of Medicine, Chi Mei Medical Center, Tainan, Taiwan; 2 Department of Health and Nutrition, Chia Nan University of Pharmacy and Science, Tainan, Taiwan; 3 Department of Intensive Care Medicine, and Chi Mei Medical Center, Liouying, Tainan, Taiwan; 4 Department of Nursing, Chi Mei Medical Center, Liouying, Tainan, Taiwan; 5 Department of Nursing, Min-Hwei College of Health Care Management, Tainan, Taiwan; Queensland Institute of Medical Research, Australia

## Abstract

**Aim:**

This study is conducted to investigate the clinical characteristics of patients with bacteremia caused by *Aeromonas* species.

**Materials and Methods:**

Patients with bacteremia caused by *Aeromonas* species during the period 2009 to 2013 were identified from a computerized database of a regional hospital in southern Taiwan. The medical records of these patients were retrospectively reviewed.

**Results:**

A total of 91 patients with bacteremia due to *Aeromonas* species were identified. In addition to 16 (17.6%) primary bacteremia, the most common source of secondary infection is peritonitis (n = 27, 29.7%), followed by biliary tract infection (n = 18, 19.8%), and SSTI (n = 12, 13.2%), pneumonia (n = 9, 9.9%), catheter-related bloodstream infection (n =  5, 5.5%), and genitourinary tract infection (n = 4, 4.4%). *A. hydrophila* (n = 35, 38.5%) was the most common pathogen, followed by *A. veronii* biovar *sobria* (n = 31, 34.1%), *A. caviae* (n = 14, 15.4%), and *A. veronii* biovar *veronii* (n = 9, 9.9%). Forty-three (47.3%) patients were classified as healthcare-associated infections (HCAI) causes by *Aeromonas* species, and patients with HCAI were more likely to have cancer, and receive immunosuppressant than patients with community-acquired bacteremia. The overall outcomes, including rate of ICU admission, acute respiratory failure, and mortality were 33.3%, 28.6%, and 23.1%, respectively. Multivariate analysis showed that the in-hospital day mortality was significantly associated only with underlying cancer (*P* <.001), and initial shock (*P* <.001).

**Conclusions:**

*Aeromonas* species should be considered one of the causative pathogens of healthcare-associated bacteremia, especially in immunocompromised patients. In addition, it can be associated with high fatality. Cancer and initial shock were the poor prognostic factors.

## Introduction

*Aeromonas* species, Gram-negative, rod-shaped bacteria are ubiquitous in aquatic environment, such as fresh or brackish water, sewage, soil, and tap water in temperate or subtropical countries [1], [2]. Southern Taiwan locates in subtropical area and it becomes an *Aeromonas*- prevalent region [3]–[6]. A recent study showed that the average annual incidences of bacteremia due to *Aeromonas* spp. was 76 cases/million inhabitants between 2008 and 2010, which was higher than those in western countries [6]. Despite the gastrointestinal tract is the most common site of infections caused by *Aeromonas* spp. [1], [2], extra-intestinal *Aeromonas*-associated diseases such as empyema, urinary tract infections, biliary tract infections, peritonitis, and skin and soft-tissue infections have also been reported [7]–[14]. Moreover, bacteremia is another common type of infection caused by *Aeromonas* species [3]–[6], [15]–[20]. In contrast to previous belief, more and more cases with *Aeromonas* bacteremia developed in the setting of healthcare-associated infections [18], [20]. In this study, we investigated the clinical characteristics of bacteremia due to *Aeromonas* species, especially in the setting of nosocomial infections, in a regional hospital in southern Taiwan over a 5-year period and assessed the risk factors associated with mortality due to this disease.

## Methods

### Hospital setting and patient selection

This study was conducted at the Chi Mei Medical Center, a 900-bed hospital located in southern Taiwan. Patients with positive blood cultures for *Aeromonas* species between January, 2009 and September, 2013, were identified from the hospital’s computerized database. The medical records of all patients with bacteremia due to *Aeromonas* species were retrospectively reviewed and the following information was collected: age, gender, underlying conditions (history of immunosuppressant drug use, diabetes mellitus, liver cirrhosis, end-stage renal disease, and active cancer), laboratory data, microbiological findings, antimicrobial susceptibility test results, and patient outcome. The data was collected on a routine basis and the analysis was carried out retrospectively. The records and information of patient were anonymized and de-identified prior to analysis. Therefore, no informed consent was required and it was specifically waived by Institution Review Board. Ethics approval was obtained from Institution Review Board of Chi Mei Medical Center.

### Bacterial isolates and antimicrobial susceptibilities

Blood were inoculated into BACTEC culture bottles using the BACTEC 9240 system (Becton Dickinson, Cockeysville, MD, USA). Sputum culture specimens were processed on blood agar, chocolate agar, and eosin methylene blue agar media (Becton Dickinson). Gram-negative isolates that tested positive for cytochrome oxidase, glucose fermentation, citrate usage, indole production, and ornithine decarboxylase were classified as *Aeromonas* species. All strains were identified to the species level by conventional methods and were further verified by the API-20E System (bioMérieux Vitek Inc, Hazelwood, MO, USA), the ID 32 GN System (bioMérieux Vitek Inc), or the Vitek 2 ID-GNB identification card (bioMérieux Inc, Durham, NC, USA). Susceptibilities of these isolates to a battery of antimicrobial agents were determined using the disk diffusion method as described by the Clinical and Laboratory Standards Institute [21].

### Definitions

The diagnosis of infection focus of bacteremia was made based on clinical, bacteriological, and radiological investigations. Peritonitis was diagnosed in patients with clinical peritoneum inflammation plus positive culture of ascites. Biliary tract infections were diagnosed in patients with clinical hepatobiliary tract inflammation plus positive bile cultures. The bile specimens were collected by percutaneous transhepatic cholangiodrainage, percutaneous gallbladder drainage, or operation. Catheter-related bloodstream infection was defined as a positive semi-quantitative tip culture (≧15 colony-forming units), bacteremia, and/or high clinical suspicion; pneumonia was defined as a positive culture for *Aeromonas* spp. in purulent sputum samples and the presence of newly developed lung infiltrates; Genitourinary tract infection (UTI) was defined as positive urine culture with growth of ≥ 10^5^ CFU/ml and pyuria; and skin and soft tissue infection (SSTI) was defined as clinical soft tissue inflammation plus positive soft tissue or pus culture and bacteremia. If no primary focus could be identified, the bacteremia was classified as primary. Shock was diagnosed in patients with a systolic blood pressure < 90 mm Hg or in patients who required inotropic agents to maintain blood pressure. In-hospital mortality was defined as death due to any cause during hospitalization. Bacteremia was classified as healthcare-associated infections (HCAI) in patients who acquired the disease during the course of treatment for other conditions within a healthcare setting [22]. Otherwise, bacteremia was classified as community acquired. Polymicrobial infections were classified if the patients whom had other non-*Aeromonas* pathogens grew from blood samples. Inappropriate usage of antibiotics was defined as that the clinical isolates were in vitro resistant to used antimicrobial agents.

### Statistical analysis

Continuous variables are expressed as means ± standard deviations. Comparisons between each variable/category were using the chi-square test or one-way analysis of variance, as appropriate. A multivariate stepwise logistic regression model was used to identify risk factors for mortality. All statistical analyses were conducted using the statistical package SPSS for Windows (Version 19.0, SPSS, Chicago, Il, USA), and a *P* value <.05 was considered to show statistical significance.

## Results

### Clinical characteristics

The clinical characteristics of the 91 patients with bacteremia caused by *Aeromonas* species are summarized in Table 1. The patients ranged in age from 10 to 98 years (mean, 64.3. years) and half of them were classified as elderly patient with age ≥ 65 years. In addition to 16 (17.6%) primary bacteremia, the most common source of secondary infection is peritonitis (n = 27, 29.7%), followed by biliary tract infection (n = 18, 19.8%), and SSTI (n = 12, 13.2%), pneumonia (n = 9, 9.9%), catheter-related bloodstream infection (n =  5, 5.5%), and genitourinary tract infection (n = 4, 4.4%). *A. hydrophila* (n = 35, 38.5%) was the most common pathogen, followed by *A. veronii* biovar *sobria* (n = 31, 34.1%), *A. caviae* (n = 14, 15.4%), and *A. veronii* biovar *veronii* (n = 9, 9.9%). Cancer (n =  40, 44.0%) was the most common underlying disease with lung cancer (n = 16) being the most common types of cancer. Liver cirrhosis (n =  36, 39.6%) was the second most common underlying disease. Fever, and shock was the initial presentation in 69 (75.8%) and 25 (27.5%) patients, respectively. Of the patients who had polymicrobial bacteremia, *E. coli* was the most common co-pathogen (n = 13), followed by *Klebsiella pneumoniae* (n = 8), enterococci (n = 3), *Acinetobacter* spp. (n = 2), *Morganella morganii* (n = 2), *Citrobacter freundii* (n = 1), *Pseudomonas aeruginosa* (n = 1), and *Enterobacter* species (n = 1).

**Table 1:** Clinical characteristics of 91 patients with bacteremia caused by *Aeromonas* species.

	No (%) of all patients (n = 91)
Age, years (mean±SD)	64.3 ± 15.6
Age ≥ 65 years, no. (%)	48 (52.7)
Male, no. (%)	64 (70.3)
Healthcare-associated infections, no. (%)	43 (47.3)
Primary bacteremia, no. (%)	16 (17.6)
Secondary bacteremia, no. (%)	75 (83.4)
Source of infection	
Primary peritonitis	27 (29.7)
Biliary tract infection	18 (19.8)
Skin and soft tissue infection	12 (13.2)
Pneumonia	9 (9.9)
Central venous catheter-related	5 (5.5)
Genitourinary tract infection	4 (4.4)
*Aeromonas* species	
* A. hydrophila*	35 (38.5)
* A. veronii* biovar *sobria*	31 (34.1)
* A. caviae*	14 (15.4)
* A. veronii* biovar *veronii*	9 (9.9)
* Aeromonas* spp.	2 (2.2)
Underlying condition no. (%)	
Active cancer	40 (44.0)
Liver cirrhosis	36 (39.6)
Diabetes mellitus	26 (28.6)
End-stage renal disease	1 (1.1)
Receiving immunosuppressant drugs	16 (17.6)
Initial presentation, no. (%)	
Fever	69 (75.8)
Shock	25 (27.5)
Laboratory findings (mean ± SD)	
White blood cell count (cell/uL)	8580.2 ± 6038.0
Neutrophil cell count (cell/uL)	7328.1 ± 5837.6
Hemoglobin (g/dL)	11.5 ± 2.2
Platelet cell count (cell/uL)	129700 ± 94400
Aspartate transaminase (IU/L)	95.7 ± 212.5
Total-bilirubin (mg/dL)	4.6 ± 5.7
Albumin (g/dL)	2.7 ± 0.6
Urea nitrogen (mg/dL)	22.8 ± 16.8
Serum creatinine (mg/dL)	1.3 ± 0.9
C-reactive protein (mg/L)	47.5 ± 67.6
Polymicrobial infection, no. (%)	26 (28.6)
Initial inappropriate antibiotic, no. (%)	30 (33.0)
Outcome, no. (%)	
Intensive care unit admission	30 (33.0)
Mechanical ventilation	26 (28.6)
In-hospital mortality	21 (23.1)

### Comparison between patients with healthcare-associated infection (HCAI) and community-acquired infection (CAI)

In this study, 43 (47.3%) patients were classified as HCAI causes by *Aeromonas* species. Table 2 summarized the comparison between patients with healthcare-associated bacteremia and community-acquired bacteremia caused by *Aeromonas* species. We found patients with HCAI were more likely to have cancer, and to receive immunosuppressant than patients with CAI. Furthermore, the outcome including ICU admission, acute respiratory failure and in-hospital mortality were significant higher among patients with HCAI than CAI (all *P* < 0.05).

**Table 2:** Comparison of 43 patients and 48 patients with healthcare-associated and community-acquired bacteremia caused by *Aeromonas* species.

	No (%) of patients with healthcare-associated bacteremia (n = 43)	No (%) of patients with community-acquired bacteremia (n = 48)	*P* value
Age ≥ 65 years, no. (%)	23 (53.5)	25 (52.1)	1.0
Male, no. (%)	31 (72.1)	33 (68.8)	0.82
Primary bacteremia, no. (%)	4 (9.3)	12 (25.0)	0.06
Secondary bacteremia, no. (%)	39 (90.7)	36 (75.0)	0.06
Source of infection			0.77
Peritonitis	16 (37.2)	11 (22.9)	
Biliary tract infection	6 (14.0)	12 (25.0)	
Skin and soft tissue infection	3 (7.0)	9 (18.8)	
Pneumonia	5 (11.6)	4 (8.3)	
Central venous catheter-related	5 (11.6)	0 (0.0)	
Genitourinary tract infection	4 (9.3)	0 (0.0)	
*Aeromonas* species			0.19
* A. hydrophila*	14 (32.6)	21 (43.8)	
* A. veronii* biovar *sobria*	14 (32.6)	17 (35.4)	
* A. caviae*	9 (20.9)	5 (10.4)	
* A. veronii* biovar *veronii*	5 (11.6)	4 (8.3)	
* Aeromonas* spp.	1 (2.3)	1 (2.1)	
Underlying condition no. (%)			
Active cancer	27 (62.8)	13 (27.1)	**0.001**
Liver cirrhosis	16 (37.2)	20 (41.7)	0.67
Diabetes mellitus	11 (25.6)	15 (31.3)	0.64
End-stage renal disease	0 (0.0)	1 (2.1)	1.0
Receiving immunosuppressant	15 (34.9)	1 (2.1)	**<0.001**
Polymicrobial infection, no. (%)	14 (32.6)	12 (25.0)	0.49
Initial inappropriate antibiotic, no. (%)	14 (32.6)	15 (31.2)	1.0
Outcome, no. (%)			
Intensive care unit admission	19 (44.2)	11 (22.9)	**0.04**
Mechanical ventilation	18 (41.9)	8 (16.7)	**0.01**
In-hospital mortality	16 (37.2)	5 (10.4)	**0.003**

### Outcome analysis

Clinical outcomes, including rate of ICU admission, acute respiratory failure, and mortality were 33.3%, 28.6%, and 23.1%, respectively. In-hospital mortality was significantly associated with nosocomial infection, cancer, receiving immunosuppressant drugs, and shock as the initial presentation (Table 3). In contrast, overall mortality was not associated with age, gender, or other underlying conditions, such as diabetes mellitus, end-stage renal disease, polymicrobial infection or initial inappropriate antibiotic therapy. Multivariate analysis showed that the in-hospital mortality was only significantly associated with underlying cancer (95% confidence interval (CI), 0.13–0.43, *P* <.001), and initial shock (95% confidence interval (CI), 0.28–0.63, *P* <.001).

**Table 3:** Prognostic factors associated with in-hospital mortality.

Variables	No (%) of mortality (n = 21)	No (%) of survivor (n = 70)	Univariate	Multivariate	
			P value	P value	95% CI
Elderly (Age≧ 65 years)	11 (52.4)	37 (52.9)	1.0		
Male	16 (76.2)	48 (68.6)	0.59		
Primary bacteremia	1 (4.8)	15 (21.4)	0.10		
Nosocomial infection	16 (76.2)	27 (38.6)	**0.003**		
Diabetes mellitus	6 (28.6)	20 (28.6)	1.0		
Cirrhosis	10 (47.6)	26 (37.1)	0.45		
End stage renal disease	0 (0.0)	1 (1.4)	1.0		
Cancer	17 (81.0)	23 (32.9)	**<0.001**	**<0.001**	**0.13**–**0.43**
Using immunosuppressant	9 (42.9)	7 (10.0)	**0.002**		
Initial shock	14 (66.7)	11 (15.7)	**<0.001**	**<0.001**	**0.28**–**0.63**
Polymicrobial infection	6 (28.6)	20 (28.6)	1.0		
Initial inappropriate antibiotic	9(42.9)	21 (30.0)	0.42		

### Antimicrobial susceptibilities

The results of in vitro susceptibility testing of clinical *Aeromonas* isolates against various antimicrobial agents are shown in Table 4. More than 98% of clinical isolates were susceptible to amikacin and imipenem, and more than 90% of clinical isolates were susceptible to ceftriaxone, ceftazidime, cefepime, ciprofloxacin, and gentamicin. In contrast, more than 90% of the isolates were not susceptible to ampicillin or cefazolin.

**Table 4:** Rates of *Aeromonas* species that were not susceptible to 12 antimicrobial agents by the disk diffusion method.

	No of isolate (%) (n = 91)
Ampicillin	90 (98.9)
Ampicillin-sulbactam	67 (73.6)
Cefazolin	86 (94.5)
Cefuroxime	13 (14.3)
Ceftriaxone	7 (7.7)
Ceftazidime	7 (7.7)
Cefepime	6 (6.6)
Piperacillin-tazobactam	15 (16.5)
Imipenem	1 (1.1)
Ciprofloxacin	3 (6)
Gentamicin	3 (3.3)
Amikacin	0 (0.0)

## Discussion

This study investigating 91 patients with *Aeromonas* bacteremia at a hospital in southern Taiwan during a 5-year period had several significant findings. The most striking finding is that about half of *Aeromonas* bacteremia developed in the setting of HCAI. In the Caribbean Islands, Hochedez et al demonstrated that 10 (27%) of 37 *A. hydrophila* bacteremia [18]. In another study [20], Chuang et al found that HCAI was noted in 26 (30%), 10 (45%), and 7 (16%) of 87 *A. hydrophila*, 22 *A. caviae*, and 45 *A. veronii* biovar *sobria* bacteremia episodes, respectively. In addition, the present work showed the clinical characteristics of the patients with HCAI that most of the patients in this subgroup had variable immunocompromised conditions, including cancer (n = 27, 62.8%), liver cirrhosis (n = 16, 37.2%), receiving immunosuppressant (n = 15, 34.9%), and diabetes mellitus (n = 11, 25.6%). It suggests that despite HCAI is unusual for *Aeromonas* species, clinicians should consider *Aeromonas* species can be one of the possible pathogens causing healthcare-associated bacteremia, especially in immunocompromised patients, such as patient with cancer or undergoing immunosuppressant. Most important of all, the overall in-hospital mortality of *Aeromonas* related HCAI was 37.2%, which was significant higher in the setting of community-acquired infection (P = 0.003), and the poor outcome may be due to the relative immunocompromised condition in this subgroup.

Overall, we found that approximately 50% of the patients with *Aeromonas* bacteremia were older than 65 years and that most of them had various underlying diseases, such as cancer and liver cirrhosis. Moreover, more than 75% of the cases of *Aeromonas* bacteremia developed in patients with immunocompromised conditions, which was defined as the patient had any one of the following conditions – cancer, liver cirrhosis, diabetes mellitus, end-stage renal disease, and receiving immunosuppressant. Similar findings have been reported in previous reports [16]–[20]. Therefore, physicians should be aware that immunocompromised patients or elderly patients at risk of developing *Aeromonas* bacteremia.

In this study, the morbidity and mortality rates associated with *Aeromonas* bacteremia were relatively high. Of the 91 patients who developed bacteremia due to *Aeromonas* species during the study period, about 30% required ICU admission and the use of mechanical ventilation. Furthermore, more than 20% of the patients died. These findings are consistent with those reported in previous studies [17]–[20]. Moreover, we found that active cancer, and shock as the initial presentation were independent risk factors for death due to *Aeromonas* bacteremia.

In this study, *A. hydrophila* was the most common *Aeromonas* species, followed by *A. caviae*. We found similar findings in our previous studies on biliary tract infections, urinary tract infections, and pneumonia due to *Aeromonas* [8], [9], [13], [14]. In contrast to previous reports that Lamy et al. found that *A. caviae* and *A. veronii* were the most common *Aeromonas* species causing bacteremia and gastroenteritis in France [23], and *A. caviae* is the most frequent pathogen causing Aeromonas bacteremia in Japan [15]. Even in the other region of Taiwan, the epidemiological finding is different that *A. hydrophila* was the most common *Aeromonas* species causing bacteremia, followed by *A. veronii* biovar *sobria* [18]. Therefore, more epidemiological studies are needed to establish the bacteriology of different types of *Aeromonas* infections in different regions.

The antibiotic susceptibility patterns of the clinical isolates in this study were similar to those reported previously [2]. Although most of the isolates were not susceptible to ampicillin or first-generation cephalosporins, more than 90% of clinical isolates were susceptible to at least third-generation cephalosporins, aminoglycosides, fluoroquinolones, and imipenem. However, the intrinsic chromosomally encoding beta-lactamases in clinical isolates of *Aeromonas* species may confound the therapeutic efficacy of in vitro active antibiotics. Although it suggests that third- or fourth-generation cephalosporins as well as fluoroquinolones may be considered the antibiotic treatments of choice for patients with severe *Aeromonas* bacteremia based on the in vitro studies, clinicians still need to keep in mind one therapeutic dilemma - the complexity of in vitro-in vivo correlation in antimicrobial treatment of *Aeromonas* infections. This study had one major limitation about the identification of *Aeromonas* species. *Aeromonas* species identified by commercial systems in the present work were not reliable enough and DNA sequencings of partial rpoD, gyrB or rpoB genes were recommended for species identification. However, the clinical isolates in this study was not kept for further identification. Further study using advanced identification method is warranted for better understand the clinical characteristics of each *Aeromonas* species.

In conclusion, healthcare-associated bacteremia caused by *Aeromonas* species is not uncommon, especially in immunocompromised patients, and can be associated with high fatality. Overall, cancer and shock as the initial presentation are significant risk factors for mortality of patients with *Aeromonas* bacteremia.
